# Comprehensive Assessment of Selected Immune Cell Subpopulations Changes in Chemotherapy-Naïve Germ Cell Tumor Patients

**DOI:** 10.3389/fonc.2022.858797

**Published:** 2022-03-11

**Authors:** Katarina Kalavska, Zuzana Sestakova, Andrea Mlcakova, Paulina Gronesova, Viera Miskovska, Katarina Rejlekova, Daniela Svetlovska, Zuzana Sycova-Mila, Jana Obertova, Patrik Palacka, Jozef Mardiak, Miroslav Chovanec, Michal Chovanec, Michal Mego

**Affiliations:** ^1^ Translational Research Unit, Faculty of Medicine, National Cancer Institute, Comenius University, Bratislava, Slovakia; ^2^ Department of Molecular Oncology, Cancer Research Institute, Biomedical Research Center, Slovak Academy of Sciences, Bratislava, Slovakia; ^3^ Department of Genetics, Cancer Research Institute, Biomedical Research Center, Slovak Academy of Sciences, Bratislava, Slovakia; ^4^ Department of Laboratory Medicine, National Institute of Children's Diseases, Bratislava, Slovakia; ^5^ Department of Hematology, National Cancer Institute, Bratislava, Slovakia; ^6^ Department of Tumor Immunology, Cancer Research Institute, Biomedical Research Center, Slovak Academy of Sciences, Bratislava, Slovakia; ^7^ 1^st^Department of Oncology, Faculty of Medicine, St. Elisabeth Cancer Institute, Comenius University, Bratislava, Slovakia; ^8^ 2^nd^Department of Oncology, Faculty of Medicine, National Cancer Institute, Comenius University, Bratislava, Slovakia; ^9^ Department of Oncology, National Cancer Institute, Bratislava, Slovakia

**Keywords:** germ cell tumors, biomarkers, innate immune cells, adaptive immune cells, tumor burden, patient outcome

## Abstract

The pattern of immune cell distribution in testicular germ cell tumors (GCT) significantly differs from the immune environment in normal testicular tissues. The present study aimed to evaluate the role of different leukocyte subpopulation in GCTs. A cohort of 84 chemotherapy-naïve GCT patients was analyzed. Immunophenotyping of peripheral blood leukocyte subpopulations was carried out by flow cytometry. In addition, the data assessing the immunophenotypes and the baseline clinicopathological characteristics of the included subjects were statistically evaluated. Their prognostic value for the assessment of progression-free survival (PFS) and overall survival (OS) was estimated. The percentage of different innate/adaptive immune cell subpopulations was significantly associated with poor risk-related clinical features, including the number of metastatic sites, presence of retroperitoneal, mediastinal, lung, brain and non-pulmonary visceral metastases as well as with the S-stage and International Germ Cell Consensus Classification Group (IGCCCG) risk groups. In univariate analysis, the percentages of neutrophils, eosinophils, dendritic cells type 2, lymphocytes and T cytotoxic cells were significantly associated with PFS, while the neutrophil, non-classical monocyte and lymphocyte percentage were associated with OS. However, all these outcome correlations were not independent of IGCCCG in multivariate analysis. The data indicated a link among different innate/adaptive peripheral immune cell subpopulations in GCT patients. In addition, the association between these subpopulations and tumor characteristics was also investigated. The findings of the present study may contribute to a deeper understanding of the interactions between cancer and innate/adaptive immune response in GCT patients.

## Introduction

Testicular germ cell tumors (GCTs) represent the most common type of solid malignancy in young males between 20 and 40 years of age (1). Overall, GCTs (non-teratoma) are characterized by an unique sensitivity to cisplatin-based chemotherapy. The rate of relapse following first-line treatment is 10-15% (2). However, the efficacy of cisplatin-based chemotherapy in multiple relapsed/refractory GCTs (rGCTs) is inferior and the majority of patients suffering from this type of GCT have an extremely poor prognosis with the long-term survival rate <5% (3). Moreover, extragonadal GCTs were found to be associated with an inferior survival rate in a patients with brain metastases using a conventional dose of cisplatin-based chemotherapy (4, 5). Despite the several phase II trials evaluating new treatment options for patients with rGCT, conventional salvage curative approaches with the limited activity continue to be utilized in the treatment of these patients (6). The mechanisms underlying the pervasive growth of cancer cells in rGCT patients remain poorly understood (7).

Over the last decade, the multifactorial interaction between the immune system and cancer cells has been considered as a hallmark of cancer, including both the systemic and local inflammatory response (8, 9). Recent data support the notion that inflammation plays an important role in tumor biology, including tumor development, progression and prognosis (10). Immune cells are able to induce or promote angiogenesis, tumor growth, invasion and metastasis *via* the production of several mediators and cytokines (11, 12). Grivennikov et al. hypothesized that certain inflammatory cytokines triggered by changes in the tumor microenvironment (TME) may lead to alterations of acute phase reactants, including neutrophil and lymphocyte counts (8). To date, several studies have been published on the evaluation of the prognostic role of markers of systemic inflammation, such as leukocyte, neutrophil and thrombocyte counts, in diverse cancer types, including colorectal, gastric, bladder and kidney cancer (13–15).

The mammalian testes represent immunologically privileged sites maintaining the balance between the immune system integrity and its ability to respond to infections and inflammation. The specific immunological environment of the testes protects germ cells from autoimmune attack and dampens the inflammatory response of testicular immune cells to antigens (16, 17). Although substantial progress has been made in understanding of involvement of the immune system in GCT biology, more detailed studies are required. A limited number of studies have been published on describing the phenomenon of spontaneous testicular tumor regression, known as “burnout”. This phenomenon is thought to be linked to the host’s immune microenvironment, as well as to the amended vascularization of the tumor. However, the etiology and pathogenesis of this condition remains poorly defined (18–20). The tumor immune microenvironment of the GCTs was comprehensively evaluated in a study published by Siska et al. who indicated that advanced-stage tumors were characterized by increased regulatory T-cells, neutrophils and mast cells signatures, irrespective of the histological subtype. Elevated macrophage signatures were also described in these cases. By contrast, T-cell and natural killer (NK)-cell signatures were shown to be decreased (21). Pearce et al. assessed the specific immunological responses of patients with GCT against cancer/testis antigens (CTAs), which were predominantly mediated by strong cluster of differentiation (CD)8+ and CD4+ CTAg-specific T-cell responses (22). Several systemic inflammatory markers that are easily obtained from routine blood tests, such as leukocyte, neutrophil and thrombocyte counts, have been proposed as potentially additional predictive and prognostic markers that can be used in various malignancies. In recent years, several reports established the prognostic and the predictive role of inflammatory indices, such as the neutrophil-to-lymphocyte ratio (23–28), platelet-to-lymphocyte ratio (28, 29) and the systemic immune-inflammation index (SII) (24, 28, 30) in testicular GCTs. All these data emphasized the important role of the immune response in GCTs (29). Therefore, there is an evident need to address the influence of the immunological response on the development of GCTs, as well as on the outcome of GCT patients. The present study investigated whether the percentage of leukocyte subpopulations was associated with different clinicopathological characteristics of GCT patients. The study further assessed whether the percentage of the selected leukocyte immunophenotypes correlated with the clinical outcome of GCT patients.

## Materials and Methods

### Study Patients

The current prospective translational study was performed on 84 chemotherapy-naïve GCT patients who were treated between October 2012 and January 2021 at the National Cancer Institute and/or the St. Elisabeth Cancer Institute, Bratislava, Slovakia (**Table 1**). Informed consent forms were obtained from all participants prior to study enrollment. The present study was approved by the Institutional Review Board and Ethical Committee of the National Cancer Institute, Bratislava, Slovakia (protocol No. IZLO1; Chair: M. Mego, from 10 February 2010). The data regarding age, tumor histological subtype, clinical stage of primary disease at diagnosis, type and number of metastatic sites as well as dates of diagnosis, progression, death and last follow-up, were obtained for all patients. The clinical stage of the primary disease was classified according to the criteria in the American Joint Committee on Cancer (AJCC), 8^th^ edition (2017) (31). Peripheral blood (PB) for immunophenotyping of leukocyte subpopulations was obtained prior to the 1^st^ cycle of platinum-based chemotherapy. The patients with evidence of concomitant malignancies other than non-melanoma skin cancer reported in the previous 5 years were excluded from the study.

**Table 1:** Baseline patient´s characteristics.

Variable	*N*	%
All patients	84	100.0
**Histology**		
Seminoma	22	26.2
Non-seminoma	62	73.8
**Primary tumor localization**		
Testicular	80	95.2
Extragonadal	4	4.8
**IGCCCG risk group**		
Good risk	62	73.8
Intermediate risk	6	7.1
Poor risk	16	19.0
Stage IA and IB (adjuvant therapy)	13	15.5
**Sites of metastases**		
Retroperitoneum	62	73.8
Mediastinum	13	15.5
Lungs	18	21.4
Liver	10	11.9
Brain	2	2.4
Other	1	1.2
Visceral non-pulmonary metastases	12	14.3
**No. of metastatic site(s)**		
0 to 2	67	79.8
≥ 3	17	20.2
**Staging (UICC)**		
IA	2	2.4
IB	11	13.1
IS	5	6.0
IIA	9	10.7
IIB	16	19.0
IIC	6	7.1
IIIA	9	10.7
IIIB	9	10.7
IIIC	17	20.2

IGCCCG, International Germ Cell Consensus Classification Group; UICC, Union for International Cancer Control.

### Immune Evaluation of Leukocyte Subpopulations

**Figure 1:**
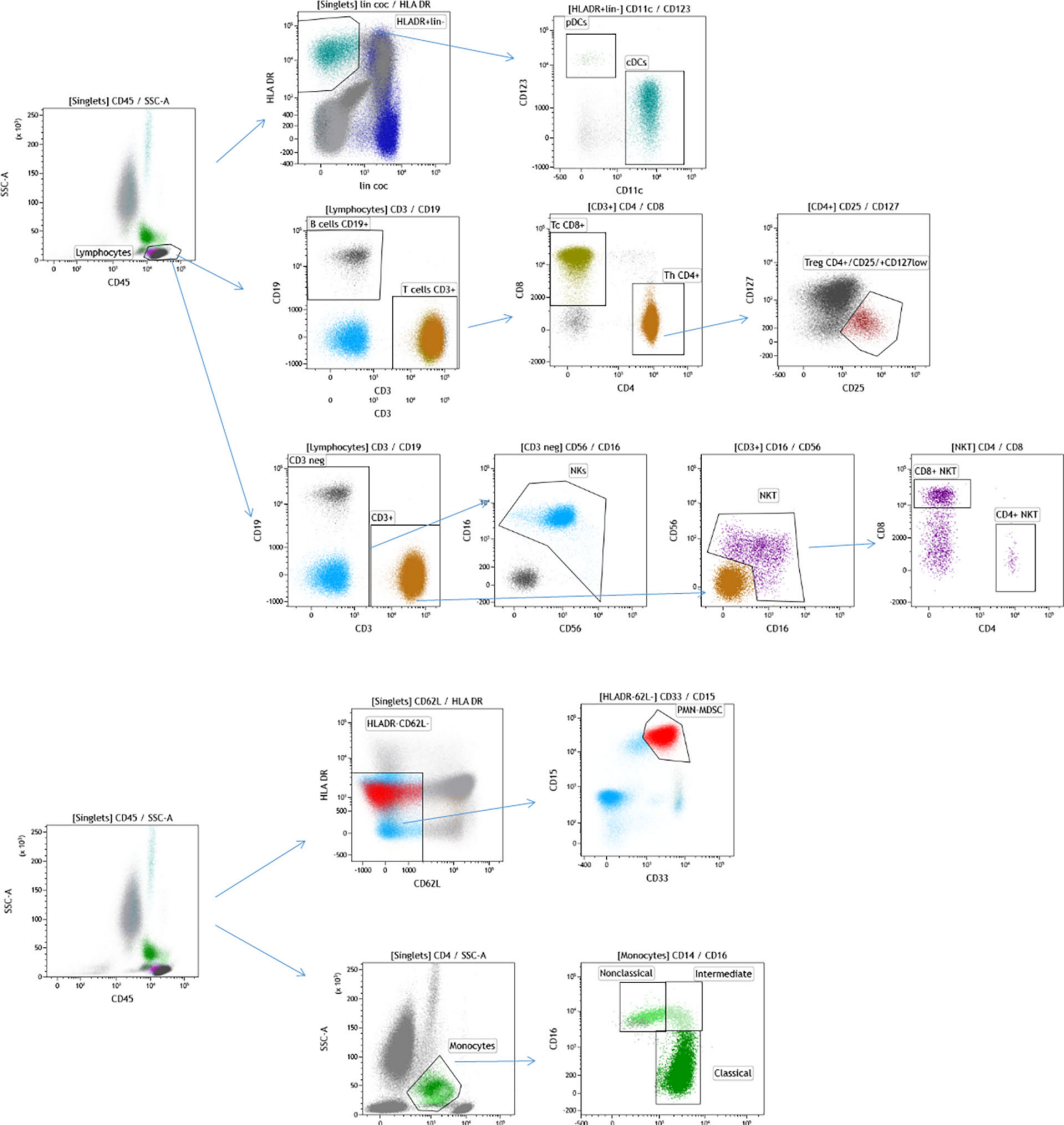
Flow cytometry gating strategy used for immunophenotyping of selected leukocyte subpopulations in the analyzed cohort of patients with GCT. The cells were initially gated by FSC and SSC following doublet exclusion using forward scatter area (FSC-A)/forward scatter height (FSC-H) (not shown). DCs were identified by CD45+ HLADR+ lin- and subsequently distinguished by CD123+ CD11c-(pDC) and CD11c+ (mDC) expression, as shown on the top of the figure. The total lymphocyte percentage was gated using a CD45/SSC plot and subsequently gating was performed for CD19+ (B cells) vs. CD3+ (T cells), CD4+ (Th cells) vs. CD8+ (Tc cells), CD56 + CD16 + CD3 (NK cells) and CD56+CD16+CD3+ (NKT cells) as well as their subpopulation (CD4+ NKT, CD8+NKT). CD4+ cells were used for the identification of CD25+ CD127-/low (Tregs). GCT, germ cell tumors; FSC, forward scatter; SSC, side scatter; CD, cluster of differentiation; HLA, human leukocyte antigen; DCs, dendritic cells; Th, T helper; Tc; T cytotoxic; NK, natural killer; Tregs, regulatory T cells. CD15 + CD33 + CD62L-HLADR-/low are polymorphonuclear myeloid-derived suppressor cells (PMN-MDSCs) and subpopulations of monocytes- CD14+CD16- (Classical monocytes), CD16+CD14- (Nonclassical monocytes) and CD14+CD16+ (Intermediate monocytes) are shown on the bottom of the figure.

From each enrolled study subject, a traumatic PB sample (1 mL) was collected at the antecubital fossa and transferred into EDTA-treated collection tubes at baseline in the morning on day -1 or 0 of the first line of chemotherapy. This sample served as the starting material for the determination of the leukocyte immunophenotype. The PB samples were processed within 24 h following collection. The analyzed leukocytes were stained with fluorochrome-conjugated antibodies obtained from BD Pharmingen. Selected leukocyte subpopulations were enumerated using flow cytometry (Becton Dickinson Canto II Cytometer) and a specific flow cytometry gating strategy was used (**Figure 1**). The antibody combinations used were as follows: 1. Basic panel—CD8 FITC (clone SK1, cat. no.: 345772, BD Biosciences, San Jose, CA 95131 USA), CD56 PE (clone MY31, cat. no.: 345810, BD Biosciences, San Jose, CA 95131 USA), CD45 PerCP Cy5.5 (clone SK3, cat. no.: 332772, BD Biosciences, San Jose, CA 95131 USA), CD19 PE-Cy7 (cat. no.: IM3628, Beckman Coulter Immunotech SAS, Marseille, France), CD3 APC (clone SK7, cat. no.: 345767, BD Biosciences, San Jose, CA 95131 USA), CD16 APC-H7 (clone 3G8, cat. no.: 560195, BD Pharmingen, San Diego, CA 92121 USA), CD4 V450 (clone RPA-T4, cat. no.: 560345, BD Biosciences, San Jose, CA 95131 USA) and CD14 HV500 (clone M5E2, cat. no.: 561391, BD Biosciences, San Jose, CA 95131 USA); 2. Regulatory T cell panel—CD3 FITC (clone SK7, cat. no.: 345763, BD Biosciences, San Jose, CA 95131 USA), CD127 PE (clone hIL-7R-M21, cat. no.: 557938, BD Pharmingen, San Diego, CA 92121 USA), CD4 PerCP Cy5.5 (clone SK3, cat. no.: 566923, BD Biosciences, San Jose, CA 95131 USA), CD25 PE-Cy7 (clone 2A3, cat. no.: 335824, BD Biosciences, San Jose, CA 95131 USA) and CD45 HV450 (clone HI30, cat. no.: 560367, BD Biosciences, San Jose, CA 95131 USA); 3. Dendritic cell panel—Lin FITC (lineage cocktail 2 FITC, cat. no.: 643397, BD Biosciences, San Jose, CA 95131 USA), CD1c PE (clone F10/21A3, cat. no.: 564900, BD Pharmingen, San Diego, CA 92121 USA), HLA-DR PerCP (clone L243, cat. no.: 347402, BD Biosciences, San Jose, CA 95131 USA), CD123 PE-Cy7 (clone 7G3, cat. no.: 560826, BD Pharmingen San Diego, CA 92121 USA), CD11c APC (clone B-Ly 6, cat. no.: 560895, BD Biosciences, San Jose, CA 95131 USA), CD16 APC-H7 (clone 3G8, cat. no.: 560195, BD Pharmingen San Diego, CA 92121 USA) and CD45 HV450 (clone HI30, cat. no.: 560367, BD Biosciences, San Jose, CA 95131 USA); and 4. Myeloid-derived suppressor cell panel—CD15 FITC (cat. no.: IM1423U, Beckman Coulter Immunotech SAS, Marseille, France), CD11b PE (cat. no.: IM2581U, Beckman Coulter Coulter Immunotech SAS, Marseille, France), HLA-DR PerCP (clone L243, cat. no.: 347402, BD Biosciences, San Jose, CA 95131 USA), CD62L PE-Cy7 (clone DREG-56, cat. no.: 565535, BD Biosciences, San Jose, CA 95131 USA), CD33 APC (clone P67.6, cat. no.: 345800, BD Biosciences, San Jose, CA 95131 USA), CD14 APC-H7 (clone MΦP9, cat. no.: 641394, BD Biosciences, San Jose, CA 95131 USA), CD66b V450 (clone G10F5, cat. no.: 561649, BD Biosciences, San Jose, CA 95131 USA) and CD45 BV510 (clone 30-F11, cat. no.: 103138, Biolegend, San Diego, CA 91121 USA).

Briefly, the cocktail of the monoclonal antibodies (4 tubes, each with 8 fluorochromes, described in detail in the section above) was incubated with 300,000-500,000 white blood cells in 200 µl for 20 min at room temperature (RT). The red blood cells were lysed and subsequently fixed by 2 mL of 1x BD FACS Lysing Solution (BD Bioscience, cat. no: 349202) during incubation for 10 min at RT. The analyzed samples were then centrifuged at 200 × g for 5 min. The evaluated samples were additionally washed twice with PBS prior to analysis. A minimum of 100,000 total leukocytes were used for evaluation on a BD FACSCanto™ II flow cytometer (Becton Dickinson, Franklin Lakes, NJ, USA). The analysis of the flow cytometry data was carried out by KALUZA software (Beckman Coulter). Size and granularity were used as criteria for the exclusion of debris by forward scatter (FSC) and side scatter, while FSC-Height and FSC-Area served for the exclusion of the doublets. The minimum number of the gated cells was 100.

### Statistical Analysis

The tabulated study data were assessed by descriptive statistical methods with median (range) for continuous variables, while the assessment of frequency (percentage) was used for categorical variables. The Kolmogorov-Smirnov test was performed in order to assess the normality of the data distribution. The data demonstrating normal distribution were analyzed by the Student’s t-test or analysis of variance, while non-normally distributed parameters were statistically evaluated by the non-parametric Mann-Whitney U-test or the Kruskal-Wallis H test. Pearson’s or Spearman’s correlation was used according to the normality of the data.

In order to perform survival analyses, the percentage of all analyzed leukocyte subpopulations were dichotomized as “low” or “high” using the median percentage of all enrolled study subjects. The median follow-up period was defined as a median observation time among all patients and among the patients who were alive at the time of their last follow-up. Progression-free survival (PFS) was calculated at the time from day one of the 1^st^ cycle of chemotherapy administration to the date of the progression of the disease or last follow-up, while overall survival (OS) was defined as the time from day one of the 1^st^ cycle of chemotherapy administration to the date of death or last follow-up. PFS and OS were assessed by the Kaplan-Meier product-limit method and compared between different groups using the log-rank test. Hazard ratios and 95% confidence intervals were estimated using logistic regression and Cox proportional hazard analysis, respectively. The multivariate Cox proportional hazard regression analysis was used to assess whether the prognostic factors associated with PFS and OS in the univariate analysis were independent of the International Germ Cell Consensus Classification Group (IGCCCG) classification system. Statistical analyses were performed using NCSS 11 Statistical Software (2016, NCSS, LLC., Kaysville, UT, USA, ncss.com/software/ncss, 8 November 2021).

## Results

### Patient Characteristics

A total of 84 chemotherapy-naive GCT patients were eligible to be enrolled in this prospective study. The baseline characteristics of all study subjects are summarized in **Table 1**. The majority of the patients exhibited primary testicular tumors. A total of 62 (73.8%) patients presented with non-seminomas and 22 (26.2%) with seminomas. The majority of the patients exhibited metastatic disease with an improved prognosis according to the IGCCCG criteria, whereas 13 out of 84 enrolled patients exhibited stage I disease and were treated with adjuvant therapy. All patients were treated with platinum-based chemotherapy and all of them received granulocyte-colony stimulating factor support (filgrastim or pegfilgrastim) following chemotherapy.

### Association Between the Percentage of Specific Innate Immune Cell Subpopulations and Clinicopathological Characteristics of the Enrolled GCT Patients

The percentage of specific innate immune cell subpopulations and their distribution with regard to the analyzed clinicopathological characteristics is summarized in **Table 2**. Univariate analysis indicated significant associations between the percentage of neutrophils and the IGCCCG risk group, the number of metastatic sites, and the presence of retroperitoneal, mediastinal, lung, brain and non-pulmonary visceral metastases. Moreover, the percentage count of neutrophils was significantly higher in patients with GCT and S-stage 3 compared with patients with S-stage 0-2 disease.

**Table 2:** Association between the percentage of innate immunity cells and the clinicopathological characteristics of the analyzed cohort of GCT patients.

Total white blood cell population (CD45+ population)	Total leukocyte subpopulations (percentage)																												Monocyte subpopulations (percentage)																																		
	Neutrophils percentage															Monocytes percentage													Classical monocytes percentage													Intermediate monocytes percentage									Non-classical monocytes percentage												
Variable	N			Mean			SEM			Median			*p*-value			N		Mean			SEM			Median			*p*-value		N		Mean			SEM			Median				*p-*value	N	Mean			SEM		Median		*p*-value	N		Mean			SEM		Median				*p-*value	
**All patients**																																																															
**Histology**																																																															
Pure seminoma	22			60.7			2.9			62.0			0.63254			22		10.0			0.7			9.4			0.39843		15		87.6			1.8			86.5				0.55907	11	4.1			0.8		3.6		0.14329	15		5.7			0.9		5.0				0.42177	
Non-seminoma or mixed GCTs	62			63.2			1.7			62.1						62		9.3			0.4			9.3					53		86.5			1.0			87.4					33	5.4			0.4		5.2			51		4.9			0.5		4.6					
**IGCCCG risk group**																																																															
Good prognosis	49			59.7			1.7			61.1			**0.00005**			49		9.7			0.5			9.4			0.12092		35		87.1			1.2			86.5				0.20119	23	4.6			0.5		3.6		0.23378	33		5.1			0.6		4.6				0.09703	
Intermediate prognosis	6			67.6			4.8			69.0						6		11.9			1.3			14.0					6		85.4			3.0			86.7					3	5.5			1.5		5.9			6		6.9			1.4		4.9					
Poor prognosis	16			76.8			2.9			78.4						16		7.9			0.8			7.5					15		88.2			1.9			90.8					9	6.2			0.9		5.5			15		3.6			0.9		2.8					
**Number of metastatic sites**																																																															
0 to 2	67			59.7			1.5			59.8			**0.00015**			67		9.4			0.4			9.3			0.73838		53		85.7			0.9			86.4				**0.01983**	35	5.1			0.4		4.4		0.65199	52		5.5			0.5		5.1				**0.00635**	
≥ 3	17			73.6			3.0			73.4						17		9.8			0.8			10.2					15		90.2			1.7			90.7					9	5.2			0.9		5.2			14		3.3			0.9		3.3					
**Retroperitoneal LN metastases**																																																															
Absent	22			58.4			2.8			57.7			**0.04037**			22		9.5			0.7			9.1			0.54158		19		86.0			1.6			84.8				0.24813	14	4.8			0.7		3.7		0.65014	19		5.1			0.8		5.0				0.58557	
Present	62			64.0			1.7			64.6						62		9.5			0.4			9.7					49		87.0			1.0			88.3					30	5.3			0.5		5.3			47		5.1			0.5		4.6					
**Mediastinal LN metastases**																																																															
Absent	71			60.1			1.5			60.4			**0.00022**			71		9.6			0.4			9.3			0.39003		57		85.9			0.9			86.4				**0.00615**	38	5.2			0.4		4.8		0.91828	55		5.5			0.4		5.0				**0.00655**	
Present	13			75.5			3.4			78.3						13		8.8			0.9			8.1					11		91.2			2.0			92.1					6	4.8			1.1		5.3			11		3.1			1.0		3.0					
**Lung metastases**																																																															
Absent	66			59.2			1.5			59.9			**0.00004**			66		9.7			0.4			9.3			0.40738		52		86.3			1.0			86.3				**0.02503**	34	5.1			0.4		4.8		0.91080	51		5.5			0.5		5.0				**0.01346**	
Present	18			74.8			2.8			74.8						18		9.0			0.8			8.8					16		88.2			1.8			91.0					10	5.1			0.8		5.2			15		3.7			0.9		3.0					
**Brain metastases**																																																															
Absent	82			62.1			1.5			61.8			**0.05281**			82		9.6			0.4			9.4			0.18672		66		86.5			0.8			86.7				**0.02682**									NA	64		5.2			0.4		4.8				**0.03003**	
Present	2			81.6			9.3			81.6						2		7.0			2.3			7.0					2		95.8			4.9			95.8														2		0.8			2.3		0.8					
**Non-pulmonary visceral metastases**																																																															
Absent	72			59.9			1.4			59.9			**000001**			72		9.6			0.4			9.4			0.32812		56		85.6			0.9			86.3				**0.00097**	38	5.1			0.4		4.8		0.51582	54		5.7			0.4		5.1				**0.00005**	
Present	12			78.4			3.4			79.4						12		8.8			0.9			7.6					12		91.9			1.9			92.5					6	5.5			1.1		5.3			12		2.3			0.9		2.1					
**S – stage**																																																															
0-2	70			59.6			1.4			59.9			**0.00002**			70		9.8			0.4			9.5			0.07567		55		86.6			1.0			86.5				0.14478	36	4.9			0.4		4.3		0.22353	53		5.1			0.5		4.9				0.14674	
3	14			77.1			3.2			79.4						14		8.1			0.8			7.4					13		87.2			2.0			90.8					8	6.0			0.9		5.7			13		4.1			0.9		3.7					
Total white blood cell population (CD45+ population)	Total leukocyte subpopulations (percentage)																																									Lymphocyte subpopulations (percentage)																					
	PMNs-MDSC percentage														Eosinophils percentage														Basophils percentage													NKT cells percentage									CD4+ NKT cells percentage												
Variable	N		Mean			SEM			Median			*p*-value			N		Mean			SEM			Median			*p*-value			N	Mean			SEM			Median			*p-*value			N	Mean		SEM			Median		*p*-value	N	Mean			SEM		Median				*p-*value		
**All patients**																																																															
**Histology**																																																															
Pure seminoma	13		0.2			0.6			0.2			0.53233			22		3.1			0.6			1.9			0.63978			22	0.7			0.07			0.6			0.89476			22	2.6		0.6			1.6		0.44796	11	0.6			0.2		0.2				0.83252		
Non-seminoma or mixed GCTs	42		0.9			0.4			0.2						62		2.4			0.3			1.6						62	0.6			0.04			0.6						60	2.2		0.4			1.3			34	0.3			0.1		0.2						
**IGCCCG risk group**																																																															
Good prognosis	30		0.4			0.4			0.2			0.63051			49		3.0			0.4			2.0			**0.01080**			49	0.7			0.04			0.6			**0,00880**			48	2.1		0.4			1.3		0.93130	24	0.4			0.1		0.2				0.75830		
Intermediate prognosis	5		0.2			1.1			0.2						6		1.9			1.0			1.6						6	0.7			0.1			0.6						6	2.2		1.0			0.9			3	1.0			0.3		0.1						
Poor prognosis	11		2.3			0.7			0.2						16		1.2			0.6			0.8						16	0.4			0.07			0.3						15	1.8		0.6			1.8			9	0.3			0.2		0.2						
**Number of metastatic sites**																																																															
0 to 2	43		0.8			0.4			0.2			1.00000			67		2.9			0.3			1.9			0.06870			67	0.7			0.04			0.7			0.10400			66	2.3		0.3			1.3		0.88833	35	0.3			0.1		0.2				0.69195		
≥ 3	12		0.2			0.7			0.2						17		1.5			0.6			1.0						17	0.5			0.08			0.5						16	2.2		0.7			1.6			10	0.5			0.2		0.2						
**Retroperitoneal LN metastases**																																																															
Absent	16		1.1			0.6			0.2			0.91141			22		2.3			0.6			1.6			0.62530			22	0.7			0.07			0.7			0.47314			22	3.0		0.6			1.4		0.31501	14	0.4			0.1		0.2				0.81567		
Present	39		0.5			0.4			0.2						62		2.7			0.3			1.9						62	0.6			0.04			0.6						60	2.0		0.3			1.3			31	0.4			0.1		0.2						
**Mediastinal LN metastases**																																																															
Absent	45		0.5			0.3			0.2			0.60784			71		2.8			0.3			1.8			0.07696			71	0.7			0.04			0.7			**0.01845**			70	2.4		0.3			1.4		0.36530	39	0.4			0.1		0.2				0.33247		
Present	10		1.7			0.7			0.2						13		1.5			0.7			1.1						13	0.5			0.09			0.4						12	1.5		0.8			1.3			6	0.3			0.2		0.1						
**Lung metastases**																																																															
Absent	43		0.7			0.4			0.2			0.34823			66		3.0			0.3			1.9			**0.00642**			66	0.7			0.04			0.6			0.09420			64	2.3		0.3			1.3		0.78802	34	0.3			0.1		0.2				0.65316		
Present	12		0.9			0.7			0.2						18		1.3			0.6			1.0						18	0.5			0.08			0.4						18	2.3		0.6			1.4			11	0.5			0.2		0.2						
**Brain metastases**																																																															
Absent	53		0.5			0.3			0.2			0.06188			82		2.6			0.3			1.7			**0.03220**			82	0.7			0.04			0.6			**0.02295**			80	2.3		0.3			1.4		**0.04093**											NA		
Present	2		7.7			1.3			7.7						2		0.2			1.8			0.2						2	0.2			0.2			0.2						2	0.1		1.9			0.1															
**Non-pulmonary visceral metastases**																																																															
Absent	47		0.3			0.3			0.2			0.07512			72		2.8			0.3			1.9			**0.00194**			72	0.7			0.04			0.7			**0.00189**			71	2.3		0.3			1.3		0.90253	39	0.4			0.1		0.2				0.59286		
Present	8		3.1			0.7			0.3						12		1.0			0.7			0.6						12	0.4			0.09			0.3						11	2.0		0.8			1.8			6	0.3			0.2		0.2						
**S – stage**																																																															
0-2	44		0.7			0.3			0.2			0.41155			70		2.9			0.3			1.9			**0.00598**			70	0.7			0.04			0.7			**0.00704**			68	2.3		0.3			1.3		0.71158	37	0.3			0.1		0.2				0.20665		
3	11		1.0			0.7			0.2						14		1.2			0.7			0.8						14	0.4			0.09			0.4						14	2.3		0.7			1.8			8	0.6			0.2		0.2						
Total white blood cell population (CD45+ population)	Lymphocyte subpopulations (percentage)																												Total leukocyte subpopulations (percentage)																						Subpopulation of DCs (percentage)												
	CD8+ NKT cells percentage														NK cells percentage														Dendritic cells (cDCs) percentage													Plasmacytoid dendritic cells (pDCs) percentage									DC2s+ percentage												
Variable		N			Mean			SEM			Median			*p*-value	N				Mean			SEM			Median			*p-*value	N			Mean			SEM			Median		*p-*value		N		Mean			SEM		Median	*p*-value	N			Mean					SEM	Median			*p*-value
**All patients**																																																															
**Histology**																																																															
Pure seminoma		11			2.6			0.6			1.9			0.53653	22				13.4			1.8			12.2			0.36522	15			1.0			0.1			0.8		0.32810		15		0.2			0.02		0.1	0.55871	14			21.8					2.4	20.3			0.93412
Non-seminoma or mixed GCTs		35			2.0			0.3			1.3				62				12.1			1.1			10.3				52			0.8			0.06			0.7				50		0.1			0.01		0.1		49			21.1					1.3	21.4			
**IGCCCG risk group**																																																															
Good prognosis		25			2.2			0.4			1.1			0.62858	49				11.0			1.2			10.0			0.11766	37			0.9			0.08			0.8		**0.00342**		36		0.2			0.01		0.2	**0.00076**	34			23.3					1.4	23.2			**0.00714**
Intermediate prognosis		3			2.2			1.2			0.5				6				24.2			3.4			22.5				5			1.1			0.2			1.2				5		0.1			0.03		0.09		5			15.3					3.7	13.0			
Poor prognosis		9			1.7			0.7			1.9				16				13.0			2.1			9.9				14			0.5			0.1			0.4				13		0.07			0.02		0.06		14			15.3					2.2	15.4			
**Number of metastatic sites**																																																															
0 to 2		36			2.2			0.3			1.2			0.98938	67				12.5			1.0			10.5			0.81946	53			0.9			0.06			0.8		**0.01358**		53		0.2			0.01		0.2	**0.00825**	51			23.3					1.1	23.1			**0.00016**
≥ 3		10			1.9			0.6			1.6				17				12.0			2.1			10.8				14			0.6			0.1			0.6				12		0.1			0.02		0.1		12			12.5					2.2	12.9			
**Retroperitoneal LN metastases**																																																															
Absent		14			2.5			0.5			1.4			0.58294	22				11.3			1.8			10.5			0.94323	19			0.8			0.1			0.8		0.49537		19		0.2			0.02		0.2	**0.00825**	18			24.3					2.0	25.3			0.07021
Present		32			2.0			0.3			1.2				62				12.8			1.1			10.9				48			0.8			0.07			0.8				46		0.1			0.01		0.1		45			20.0					1.3	17.7			
**Mediastinal LN metastases**																																																															
Absent		40			2.2			0.3			1.3			0.61316	71				12.7			1.0			10.5			0.52417	57			0.9			0.06			0.8		**0.00896**		56		0.2			0.01		0.1	**0.02360**	54			22.2					1.2	21.8			**0.03228**
Present		6			1.5			0.8			1.5				13				10.7			2.4			10.8				10			0.5			0.1			0.4				9		0.1			0.03		0.06		9			15.5					2.8	13.0			
**Lung metastases**																																																															
Absent		35			2.2			0.3			1.4			0.92818	66				11.7			1.0			10.1			0.23047	52			0.9			0.06			0.8		**0.00112**		52		0.2			0.01		0.2	**0.00086**	50			23.3					1.1	23.5			**0.00014**
Present		11			1.9			0.6			1.3				18				15.1			2.0			12.1				15			0.5			0.1			0.5				13		0.1			0.02		0.07		13			13.2					2.2	12.8			
**Brain metastases**																																																															
Absent														NA	82				12.6			0.9			10.9			0.13456	65			0.8			0.06			0.8		**0.04267**		63		0.1			0.01		0.1	**0.03012**	61			21.1					1.1	20.9			0.69503
Present															2				5.4			6.0			5.4				2			0.3			0.3			0.3				2		0.03			0.05		0.03		2			24.9					6.2	24.9			
**Non-pulmonary visceral metastases**																																																															
Absent		40			2.2			0.3			1.2			0.81940	72				12.5			1.0			10.7			0.71086	56			0.9			0.06			0.8		**0.00034**		56		0.2			0.01		0.2	**0.00029**	53			22.3					1.2	22.1			**0.03355**
Present		6			1.7			0.8			1.7				12				12.1			2.5			9.9				11			0.4			0.1			0.4				9		0.06			0.02		0.03		10			15.8					2.7	13.4			
**S – stage**																																																															
0-2		38			2.1			0.3			1.2			0.42546	70				11.8			1.0			10.3			0.33695	56			0.9			0.06			0.8		**0.03222**		55		0.2			0.01		0.2	**0.00998**	52			22.8					1.1	22.7			**0.00196**
3		8			2.2			0.7			2.0				14				15.6			2.2			12.1				11			0.6			0.1			0.6				10		0.1			0.02		0.1		11			13.8					2.5	12.8			

SEM, standard error of the mean. Values of p ≤ 0.05 are considered as significant. Significant p values are in bold. Variability of total number of examined patients samples (N) within the evaluated subpopulations were due to the individual technical limitations, including missing antibodies or bad quality of examined samples.

The analysis of the correlation between the percentage of monocytes and clinicopathological characteristics demonstrated no significant correlation. However, subgroup analysis was used to differentiate the monocyte population into classical, intermediate, non-classical and polymorphonuclear myeloid-derivate suppressor cells according to their immunophenotypes. This analysis revealed that the presence of mediastinal, lung, brain and non-pulmonary visceral metastases was significantly higher in patients with a higher percentage of classical monocytes. The higher percentage of classical monocytes was also associated with the presence of three and more metastatic sites. By contrast, the lower percentage of these immune cells in the subgroup of non-classical monocytes was significantly correlated with the presence of three and more metastatic sites, and the presence of mediastinal, lung, brain and non-pulmonary visceral metastases.

Statistically significant differences in the percentage of eosinophils were also determined in different groups of patients according to the IGCCCG criteria and the S-stage, as well as in patients with lung, brain and non-pulmonary visceral metastases.

A lower percentage of basophils was significantly correlated with poor prognosis according to the IGCCCG, S-stage, and the presence of mediastinal, brain and non-pulmonary visceral metastases. It was also found that the lower percentage of natural killer (NK) T-cells was associated with the presence of brain metastases.

A statistically significant difference was also reported between the IGCCCG risk groups, the S-stage, the number of metastatic sites, and the presence of mediastinal, lung, brain and non-pulmonary visceral metastases with regard to the percentage of dendritic cells (DCs). In addition, the percentage of CD1c-positive cells within DCs revealed a significant correlation with the same clinicopathological characteristics, with the exception of the presence of brain metastases, as well as the analysis of the whole group of DCs. It is interesting to note that the association with the same clinicopathological characteristics in the case of the percentage of DCs was also found following the analysis of the percentage of the plasmacytoid DCs (**Table 2**).

### Association Between the Percentage of Specific Adaptive Immune Cell Subpopulations and Clinicopathological Characteristics of Analyzed GCT Subjects

The percentage of specific adaptive immune cell subpopulations and their distribution with regard to the analyzed clinicopathological characteristics is summarized in **Table 3**. The analyzed data revealed that the percentage of lymphocytes was significantly associated with the IGCCCG risk group, the S-stage, as well as with the number of metastatic sites. Moreover, a highly significant effect was reported between low lymphocyte percentage and the presence of retroperitoneal lymph node, mediastinal, lung, brain and non-pulmonary visceral metastases.

Table 3Association between the percentage of the adaptive immunity cells and the clinicopathological characteristics of the analyzed cohort of the GCT patients.

**Table:** 

Total white blood cell population (CD45+ population)	Total leukocyte subpopulations (percentage)					Subpopulations of lymphocytes (percentage)																					
	Lymphocytes percentage					B cells percentage (CD14+)					T cells percentage (CD3+)					T helper cells percentage					T cytotoxic cells percentage						
Variable	N	Mean	SEM	Median	*p-*value	N	Mean	SEM	Median	*p-*value	N	Mean	SEM	Median	*p-*value	N	Mean	SEM	Median	*p-*value	N		Mean	SEM	Median	*p-*value
																									
**All patients**																										
**Histology**																										
Pure seminoma	22	27.1	2.6	25.9	0.76797	22	9.4	1.1	9.6	**0.04615**	22	74.5	2.0	75.4	0.55174	22	47.2	1.9	47.4	0.30461	22		26.0	1.5	26.2	0.74091
Non-seminoma or mixed GCTs	62	25.7	1.6	25.7		62	12.1	0.7	11.4		62	73.0	1.2	73.8		61	45.0	1.2	45.8		62		25.6	0.9	24.9	
**IGCCCG risk group**																										
Good prognosis	48	28.8	1.5	26.9	**0.00001**	49	10.9	0.8	10.8	0.72412	49	75.1	1.3	75.7	**0.02091**	48	46.5	1.3	45.8	0.21998	49		26.9	1.0	26.2	**0.03453**
Intermediate prognosis	6	18.4	4.3	12.8		6	11.6	2.3	13.4		6	61.1	3.6	62.8		6	38.3	3.7	37.5		6		19.6	2.8	22.0	
Poor prognosis	16	13.6	2.6	12.1		16	13.2	1.4	12.1		16	71.0	2.2	72.9		16	45.7	2.3	47.5		16		23.3	1.7	23.5	
**Number of metastatic sites**																										
0 to 2	67	29.0	1.3	28.3	**0.00001**	67	11.0	0.6	10.8	0.60467	67	73.7	1.1	73.9	0.82380	66	45.3	1.1	45.7	0.51281	67		26.0	0.8	26.2	0.18338
≥ 3	17	14.8	2.6	14.5		17	13.0	1.3	13.0		17	72.5	2.2	72.9		17	46.4	2.2	50.6		17		24.4	1.7	23.4	
**Retroperitoneal LN metastases**																										
Absent	22	30.4	2.6	31.3	**0.02799**	22	11.4	1.1	11.2	0.77965	22	75.2	2.0	74.0	0.61813	22	47.3	1.9	47.2	0.40914	22		25.0	1.5	25.3	0.70282
Present	62	24.6	1.5	23.7		62	11.4	0.7	10.7		62	72.8	1.2	73.8		61	44.9	1.2	45.7		62		25.9	0.9	25.2	
**Mediastinal LN metastases**																										
Absent	71	28.3	1.3	28.0	**0.00007**	71	11.3	0.6	10.8	0.77133	71	73.3	1.1	73.7	0.88690	70	44.9	1.1	45.8	0.26480	71		26.1	0.8	26.2	0.08448
Present	13	13.9	3.1	11.2		13	12.2	1.5	9.8		13	74.1	2.6	77.7		13	49.0	2.5	50.6		13		23.4	1.9	22.7	
**Lung metastases**																										
Absent	66	29.3	1.3	28.6	**0.00000**	66	10.8	0.6	10.6	0.12970	66	74.7	1.1	75.1	**0.05037**	65	46.0	1.1	45.8	0.76544	66		26.5	0.8	26.7	**0.02404**
Present	18	14.4	2.5	12.5		18	13.7	1.2	13.4		18	68.7	2.1	71.9		18	44.1	2.1	47.3		18		22.8	1.6	23.3	
**Brain metastases**																										
Absent	82	26.5	1.3	26.0	**0.05281**	82	11.4	0.6	10.8	0.70289	82	73.2	1.0	73.6	0.13456	81	45.3	1.0	45.8	0.11552	82		25.7	0.8	25.2	0.81443
Present	2	10.2	8.5	10.2		2	10.0	3.8	10.0		2	80.9	6.5	80.9		2	55.4	6.4	55.4		2		23.6	4.8	23.6	
**Non-pulmonary visceral metastases**																										
Absent	72	28.6	1.3	28.1	**0.00000**	72	11.2	0.6	11.0	0.72999	72	73.6	1.1	74.0	0.70610	71	45.0	1.1	45.2	0.12652	72		26.4	0.8	26.2	**0.01743**
Present	12	11.2	3.1	10.6		12	12.7	1.5	8.8		12	72.2	2.7	72.9		12	48.9	2.6	51.8		12		21.8	1.9	23.0	
**S – stage**																										
0-2	70	28.7	1.3	28.1	**0.00001**	70	11.1	0.6	10.8	0.78250	70	74.4	1.1	74.4	0.16203	69	46.1	1.1	45.8	0.33062	70		26.1	0.8	25.9	0.26432
3	14	13.1	2.9	10.6		14	12.9	1.4	11.7		14	68.8	2.4	71.8		14	42.6	2.4	44.2		14		23.8	1.8	23.5

**Table:** 

Total white blood cell population (CD45+ population)	Subpopulations of lymphocytes (percentage)																										
	Tregs percentage																										
Variable	N	Mean	SEM	Median	*p-*value																					
																									
**All patients**																										
**Histology**																										
Pure seminoma	22	4.0	0.3	3.8	0.81498																					
Non-seminoma or mixed GCTs	62	4.0	0.2	3.9																						
**IGCCCG risk group**																										
Good prognosis	49	4.1	0.2	4.0	0.17311																					
Intermediate prognosis	6	3.3	0.5	3.4																						
Poor prognosis	16	3.7	0.3	3.4																						
**Number of metastatic sites**																										
0 to 2	67	4.0	0.2	3.9	0.72163																					
≥ 3	17	4.0	0.3	3.9																						
**Retroperitoneal LN metastases**																										
Absent	22	4.2	0.3	3.9	0.52818																					
Present	62	3.9	0.2	3.8																						
**Mediastinal LN metastases**																										
Absent	71	3.9	0.2	3.9	0.63395																					
Present	13	4.3	0.4	3.9																						
**Lung metastases**																										
Absent	66	4.0	0.2	3.8	0.72311																					
Present	18	4.0	0.3	4.0																						
**Brain metastases**																										
Absent	82	4.0	0.1	3.9	0.86026																					
Present	2	3.8	1.0	3.8																						
**Non-pulmonary visceral metastases**																										
Absent	72	4.0	0.2	3.9	0.31562																					
Present	12	3.6	0.4	3.4																						
**S – stage**																										
0-2	70	4.0	0.2	3.8	0.96649																					
3	14	3.9	0.4	4.1																						

SEM, standard error of the mean. Values of p ≤ 0.05 are considered as significant. Significant p values are in bold. Variability of total number of examined patients samples (N) within the evaluated subpopulations were due to the individual technical limitations, including missing antibodies or bad quality of examined samples.

The analysis of the immunophenotypes belonging to adaptive immunity indicated the association between low B cell percentage and seminomatous histology. The percentage of T cells was significantly associated with the IGCCCG risk group. In addition, the presence of lung metastasis was significantly higher in patients with a lower percentage of lymphocytes. The analysis of the percentage of T helper cells and T regulatory cells (Tregs) with the clinicopathological characteristic indicated no significant association. However, the evaluation of the relationship between the percentage of T cytotoxic cells and the IGCCCG risk group revealed a significant correlation. In addition, the lower percentage of T cytotoxic cells correlated with the presence of lung and non-pulmonary visceral metastases.

### Survival Analyses

The median follow-up time was 22.6 months (range 0.2-100.4 months). In the entire cohort of the patients, 11 cases of progression (13.1%) were observed and 8 (9.5%) patients succumbed to the disease during the follow-up period.

### Prognostic Value of Innate Immune Cell Subsets in GCT Patients

**Figure 2:**
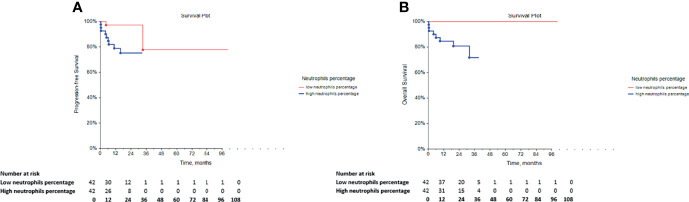
Kaplan-Meier survival curves for the assessment of the neutrophil percentage in chemotherapy-naïve patients with GCT. The log-rank test was used to assess the **(A)** PFS (*p* = 0.0084) and the **(B)** OS (*p* = 0.0022). GCT, germ cell tumors; PFS, progression-free survival; OS, overall survival.

The univariate Cox regression analysis reported a significantly longer PFS (*p* = 0.0084) and OS (*p* = 0.0022) in GCT patients with low neutrophil percentage (**Table 4** **;** **Figures 2A, B**, respectively), whereas this prognostic effect was lost following adjustment for the IGCCCG risk group in the multivariate analysis.

**Table 4:** Prognostic value of the percentage of innate immunity cells on the outcome of chemotherapy naïve GCT patients.

		Progression-free survival				Overall survival			
	N	HR	95% Low CI	95% High CI	*p*- value	HR	95% Low CI	95% High CI	*p*- value
**Neutrophils percentage**									
Low	42	0.17	0.05	0.57	**0.0084**	0.00	0.00	0.00	**0.0022**
High	42								
**Monocytes percentage**									
Low	42	1.95	0.60	6.36	0.2766	1.66	0.42	6.64	0.4809
High	42								
**Classical monocytes percentage**									
Low	34	0.45	0.12	1.68	0.2498	0.42	0.09	1.84	0.2788
High	34								
**Intermediate monocytes percentage**									
Low	22	1.93	0.20	18.6	0.5832	0.00	0.00	0.00	0.3980
High	22								
**Nonclassical monocytes percentage**									
Low	33	3.88	1.05	14.37	0.0679	6.94	1.57	30.66	**0.0363**
High	33								
**Polymorphonuclear MDSC (PMNs-MDSC) percentage**									
Low	29	1.16	0.31	4.29	0.8237	1.23	0.28	5.42	0.7861
High	26								
**Eosinophils percentage**									
Low	42	4.50	1.38	14.67	**0.0314**	3.00	0.75	11.98	0.1563
High	42								
**NKT cells percentage**									
Low	42	1.49	0.43	5.14	0.5331	1.95	0.44	8.70	0.4071
High	40								
**CD4+ NKT cells percentage**									
Low	24		0.00	0.00	0.1390	0.00	0.00	0.00	0.4227
High	21								
**CD8+ NKT cells percentage**									
Low	23	0.00	0.00	0.00	0.7217	0.00	0.00	0.00	0.4795
High	23								
**NK cells percentage**									
Low	42	1.08	0.33	3.53	0.8967	1.69	0.42	6.76	0.4640
High	42								
**Dendritic cells (cDCs) percentage**									
Low	34	1.96	0.53	7.26	0.3291	4.52	0.91	22.42	0.1306
High	33								
**Plasmocytoid dendritic cells (pDCs) percentage**									
Low	33	3.47	0.94	12.82	0.0972	4.18	0.84	20.83	0.1465
High	32								
**DC2s+ percentage**									
Low	32	9.79	2.63	36.39	**0.0075**	5.78	1.16	28.76	0.0686
High	31								

HR, hazard ratio; CI, confidence interval. Values of p ≤ 0.05 are considered as significant. Significant p values are in bold. Variability of total number of examined patients samples (N) within the evaluated subpopulations were due to the individual technical limitations, including missing antibodies or bad quality of examined samples.

Furthermore, the patients with low eosinophil percentage as well as the group of GCT patients with low DC2s exhibited significantly lower PFS compared with that of the patients with high percentages of eosinophils and DC2, respectively. In addition, survival analysis indicated a significant relationship between a low percentage of non-classical monocytes and lower OS. However, all of these associations were not independent of IGCCCG in the multivariate analysis (data not shown).

### Prognostic Value of Adaptive Immune Cell Subsets in GCT Patients

**Figure 3:**
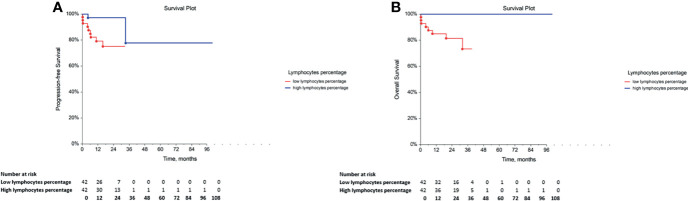
Kaplan-Meier survival curves for the assessment of the lymphocyte percentage in chemotherapy-naïve patients with GCT. The log-rank test was used to assess the **(A)** PFS (*p* = 0.0089) and the **(B)** OS *(p* = 0.0033). GCT, germ cell tumor; PFS, progression-free survival; OS, overall survival.

The assessment of the correlation between the percentage of adaptive immune cell subsets and PFS or OS indicated that GCT patients with low percentage of lymphocytes exhibited significantly lower PFS and OS **(** **Table 5**, **Figures 3A, B**, respectively. Furthermore, the comparison of PFS in patients with low and high percentages of T cytotoxic cells indicated that patients with a low percentage of these cells exhibited significantly shorter PFS compared with those with a high percentage **(** **Table 5** **)**. However, their OS was not affected **(** **Table 5** **)**. By contrast, multivariate analysis revealed that these correlations were not independent of the IGCCCG classification system (data not shown).

**Table 5:** Prognostic value of the percentage of adaptive immunity cells on the outcome of chemotherapy naïve GCT patients.

		Progression-free survival				Overall survival			
	N	HR	95% Low CI	95% High CI	*p*- value	HR	95% Low CI	95% High CI	*p*- value
**Lymphocytes percentage**									
Low	42	5.67	1.73	18.63	**0.0089**	0.00	0.00	0.00	**0.0033**
High	42								
**B cells percentage (CD14+)**									
Low	43	1.11	0.34	3.61	0.8650	3.07	0.77	12.27	0.1477
High	41								
**T cells percentage_(CD3+)**									
Low	42	1.75	0.54	5.71	0.3644	0.99	0.25	3.94	0.9838
High	42								
**T helper cells percentage**									
Low	42	0.91	0.28	2.97	0.8749	0.93	0.23	3.73	0.9213
High	41								
**T cytotoxic cells percentage**									
Low	42	4.10	1.26	13.39	**0.0497**	3.25	0.81	13.02	0.1253
High	42								
**Tregs percentage**									
Low	42	1.71	0.52	5.58	0.3844	1.60	0.40	6.41	0.5137
High	42								

HR, hazard ratio; CI, confidence interval. Values of p ≤ 0.05 are considered as significant. Significant p values are in bold. Variability of total number of examined patients samples (N) within the evaluated subpopulations were due to the individual technical limitations, including missing antibodies or bad quality of examined samples.

## Discussion

The present study performed a comprehensive analysis of the role of the percentage of the leukocyte subpopulations in PFS and OS in GCT patients. To the best of our knowledge, this is the first study that analyzed extensively the leukocyte immunophenotypes in GCT patients and their association with the clinicopathological characteristics, as well as their prognostic value in terms of PFS and OS.

Interplay between the tumor burden and immune response was reported by Whitney et al. approximately 50 years ago (32). Recent study has demonstrated that extensive disruption of hematopoiesis was driven by tumor burden (33). In this context, it is interesting to note that the previous study has assessed possible crosslink between the endogenous DNA damage levels and the changes in the immune cell repertoire. The data indicated that the DNA damage levels were correlated with the percentage of specific subpopulations of immune cells, namely NK cells, CD16+ DCs and Tregs. Based on these data, it may be assumed that the observed immune cell percentage changes reflect the characteristics of malignant processes, involving DNA damage as an important factor of TME (34). Furthermore, progressively increased frequency of bone marrow hematopoietic stem cells, multipotent progenitors and granulocyte monocyte progenitors is associated with the tumor burden. This conclusion was made on the use of mouse models of breast cancer and rhabdomyosarcoma (35–38). The fact that hematopoietic dysregulation is common in human cancer was also demonstrated in the pan-cancer study by Wu et al., who reported the elevated levels of hematopoietic stem cells, multipotent progenitors and granulocyte monocyte progenitors in the blood of patients with breast, cervical, liver, esophageal, lung, ovarian and gastrointestinal cancer (39). It is interesting to note that the ability to reorganize the immune macroenvironment in cancer is also supported by several observations when various changes in the immune cell count were reversed by surgical resection of the tumor or cytokine blockade treatment (33). Therefore, it was hypothesized that the global immune landscape across the immune cell lineages was dramatically restructured by more advanced diseases.

The vast majority of the studies evaluating immune perturbations in the context of cancer have focused on the increase of the immature and immunosuppressive myeloid populations (33). Previous studies have evaluated the prognostic or predictive value of specific markers, which were easily retrieved from blood tests performed routinely, such as those assessing the number of neutrophils, leukocytes and platelets in various malignant tumors, including GCTs (24–26, 40–43). With the exception of these markers, the alterations of several other peripheral immune lineages were involved in this process. Therefore, the present study has included much more specific subpopulations of immune cells in the overall analysis and assessed their potential link with the tumor burden.

Remodeling of the immune cell populations was associated with the tumor burden incidence. Moreover, it was most prominently characterized by increased frequencies of neutrophils and monocytes and the reduction in the percentages of DCs, B cells and T cells in the periphery of tumor-burdened hosts (33). These data are in concordance with the results obtained in the present study, where a higher neutrophil percentage was significantly associated with the presence of retroperitoneal and mediastinal lymph node metastases, as well as with the presence of lung, brain and non-pulmonary visceral metastases. Higher percentage of neutrophils were also associated with poor prognosis, a higher number of metastatic sites and a higher S-stage. These associations support the ability of the neutrophils to induce tumor cell trans-endothelial migration and metastasis (44). Furthermore, decreased levels of lymphocytes were associated with the presence of retroperitoneal lymph node metastases, mediastinal lymph node, lung, brain and non-pulmonary visceral metastases. In addition, the data demonstrated correlations among low lymphocyte percentage and poor prognosis, a higher number of metastatic sites and advanced S-stage. It is interesting to note that the increased levels of cytotoxic T cells were described in patients with poor prognosis according to the IGCCCG classification, and the presence of lung and non-pulmonary visceral metastases. These results also support the implication of the pro-inflammatory state noted in the tumor burden, which was supported by the interconnection between high neutrophil percentage and decreased percentage of lymphocytes and activated T cells (27, 45–47). In addition, survival analyses revealed that the percentage of neutrophils and lymphocytes could be used as a prognostic factor for PFS and OS, but not independently of the IGCCCG classification, in an analyzed cohort of chemotherapy-naïve GCT patients. Similar findings were also presented by Ribnikar et al. (27).

Moreover, the present study indicates that decreased eosinophil and basophil percentage were found predictors for more advanced and metastatic disease. The role of eosinophils in cancer is uncertain. Recent data have indicated that eosinophils could infiltrate the TME, by inducing cytotoxic effects on cancer cells or, alternatively, by secretion of pro-angiogenic and matrix-remodeling soluble mediators. The latter molecules may directly regulate tumor progression as either antitumorigenic or pro-tumorigenic factors. By contrast, eosinophils have been shown to display regulatory functions towards other immune cell subsets following activation of DCs by cytokine release from eosinophil granules (48, 49). This, in turn, recruits T cells and alternates the TME vasculature, which indirectly shapes the TME (48, 49). The antitumorigenic role of eosinophils is supported by the majority of studies using murine experimental models, as well as human studies. In clinical context, both peripheral and tumor-associated eosinophils were associated with improved prognosis in the majority of cancer types (50).

Furthermore, accumulating evidence has shown the immune/inflammation-related role of basophils in the tissue microenvironment surrounding a tumor (51–53). Basophils affect the TME particularly *via* the secretion of granules, such as histamine, cytokines and lipid inflammatory mediators that polarize the immune reaction response for immunoglobulin E production (54). The association between the peripheral basophils and certain solid tumors was demonstrated in both murine experimental models of cancer and primary human tumors (55). A protective role of basophils was demonstrated by several groups. Wang et al. reported the correlation between a low count of circulating basophils and the increased number of pulmonary metastases using a mouse model of breast cancer (56). A similar association was observed in our cohort of patients, where basopenia was associated with the increased presence of mediastinal, brain and non-pulmonary visceral metastases. Moreover, an association between the basopenia and poor prognosis, as well as with the higher stage of disease, was found. Poor prognosis was defined according to the IGCCCG classification. Previously published data have also suggested that basopenia appears to be linked with aggressive disease (54) and poor prognosis in cancer patients (52, 54).

The present study has also confirmed significantly increased levels of classical monocytes in patients with advanced GCT disease (association with number of metastatic sites, presence of mediastinal, lung, brain and non-pulmonary visceral metastases, as well as higher S-stage of disease) and poor prognosis. Interestingly, the inverse association was described between non-classical monocytes and poor disease characteristics in analyzed cohort. Hence, presence of mediastinal, lung, brain and non-pulmonary visceral metastases as well as higher number of metastatic sites were significantly associated with decreased non-classical monocytes level. Monocytes represent a heterogenic population of immune cells and are divided into the following three subsets according to their specific surface markers: classical (~85%), intermediate (~5%) and non-classical (~10%) monocyte population (57). Recent studies have reported that monocytes (classical and non-classical subpopulations) are extensively implicated in tumor development and progression by regulating tumor growth, antitumor immunity, angiogenesis and metastatic spread, when recruitment of monocytes was described during the establishment of distal metastases (58, 59). However, much less is known regarding the ability of tumor cells to induce alterations in monopoiesis and circulating monocytes (60). Increased levels of peripheral blood monocytes in cancer have been found in both humans and murine models (61–63). These levels were associated with a worse disease prognosis (62, 64–66). The elevated monocyte counts may occur due to raised mobilization from the bone marrow or during enhanced monopoiesis, while both of these processes are noted in the development of cancer (60). It was also shown that the number of monocytes characterized by the CD14^+^ human leukocyte antigen (HLA)-DR^low^ immunophenotype was elevated according to the tumor stage and poor survival (67, 68).

These data could be used for clarification of the link between increased classical monocyte percentage, representing the major subset on monocytes, and tumor burden. Several studies, including study published by Olingy et al. suggest that classical and non-classical monocytes in mouse model and human are counterparts (59), what is in accordance with our data. This trend, was also reported by Valdes-Ferrada et al. who showed that level of non-classical monocytes was significantly decreased after the first cycle of neoadjuvant chemotherapy (NAC) and tended to increase during the 6th cycle, while the opposite pattern was displayed in population of classical monocytes. Additionally, the opposite distribution was also showed for classical and non-classical monocytes across NAC (69). Whereas, classical monocytes are believed to be robustly recruited to primary tumors and metastatic sites, non-classical monocytes display much lower levels of recruitment (70).

In addition, the data obtained in the current analysis demonstrated low percentage of DCs in patients with advanced disease and poor prognosis. DCs represent a complex network of antigen-presenting cells that create a link between the innate and adaptive immunity. In cancer, DCs process tumor-derived antigens and present them to T cells. However, an immunosuppressive milieu in tumors may result in the inactivation of DCs and NK cells, as well as the formation of functional deficiencies in these cells. This state may subsequently result in the inhibited capacity of the host immune system to mount an effective anti/tumor T cell response (71). Decreased levels or absence of DCs or NK cells during tumor progression were observed in multiple tumor types, including renal adenocarcinoma (72) or non-small cell lung carcinoma (NSCLC) (71).

However, no correlation was determined with regard to the NK cell subpopulation and the incidence of cancer. Platonova et al. reported that NK cell perturbations were dependent on the TME specifications. No phenotypic alterations of peripheral NK cells were determined between the patients with NSCLC and healthy subjects. However, the subsequent *ex vivo* incubation of NK cells, which were isolated from these patients, with tumor cells led to the reduction of the expression levels of the NK cell receptor and to the impaired degranulation (73).

The present study exhibits certain limitations, which were associated with the limited number of patients included. In addition, the analyzed cohort consisted of a relatively low number of patients with brain metastases and a low number of events were noted in certain analyzed subgroups (especially mortality after stratification for individual clinical categories). Moreover, the immunophenotype of the PB cells was assessed in relation to the patient/tumor characteristics and the data indicated that it did not ultimately reflect the immune TME, but it was rather a manifestation of the cancer disease. Other limitations of the present study include the lack of assessment of the competence or function of selected leukocyte subpopulations. Specifically, only their percentages were included. Ultimately, a larger data set is required to exactly determine the nature of selected leukocyte subpopulation changes and to clarify the role of the immune system in the development of GCTs.

In conclusion, it has been postulated that the percentage of the selected leukocyte subpopulations is significantly associated with several clinicopathological characteristics. Moreover, it has been shown to be associated with more advanced and metastatic disease. Based on the aforementioned data, it may be inferred that the observed correlations represent the consequences of more aggressive disease and reflect the advanced tumor burden. In this state, advanced disease causes changes to the innate and adaptive immune cells, which consequently dampens antitumor activity. However, further research is required to fully elucidate these interactions.

## Data Availability Statement

The original contributions presented in the study are included in the article/supplementary material. Further inquiries can be directed to the corresponding author.

## Ethics Statement

The studies involving human participants were reviewed and approved by The Institutional Review and Ethical Committee of the National Cancer Institute, Bratislava, Slovakia, approved the protocol (No. IZLO1; Chair: M. Mego, from 10 February 2010). The patients/participants provided their written informed consent to participate in this study.

## Author Contributions

KK, MM, and ZS conceived and designed the study. KK, ZS, AM, VM, KR, DS, ZS, PP, JO, JM, MicC, and MM acquired the data. KK, MM, and ZS analyzed and interpreted the data. KK and MM performed statistical analysis. KK and MM wrote the first draft of the manuscript, and ZS, AM, MicC, and MirC critically revised the manuscript. KK, MM, and MirC obtained funding. DS contributed administrative and technical support. MM supervised the whole study. All authors contributed to the article and approved the submitted version.

## Funding

This work was supported by the VEGA Grant Agency of the Slovak Republic (grant nos. 1/0043/18, 1/0327/19, 1/0349/21, and 2/0053/19), The Slovak Research and Development Agency (grant nos. APVV-15-0086, APVV-17-0384, APVV-19-0411, and APVV-20-0158) and The Integrated Infrastructure Operational Program for the Project: Systemic Public Research Infrastructure—Biobank for Cancer and Rare Diseases, ITMS: 313011AFG5; co-financed by the European Regional Development Fund.

## Conflict of Interest

The authors declare that the research was conducted in the absence of any commercial or financial relationships that could be construed as a potential conflict of interest.

## Publisher’s Note

All claims expressed in this article are solely those of the authors and do not necessarily represent those of their affiliated organizations, or those of the publisher, the editors and the reviewers. Any product that may be evaluated in this article, or claim that may be made by its manufacturer, is not guaranteed or endorsed by the publisher.
